# Development and validity test of impression management efficacy scale based on self-presentation behavior of Chinese youth on social media

**DOI:** 10.3389/fpsyg.2025.1494083

**Published:** 2025-01-30

**Authors:** Yixuan Liu, Ke Lei

**Affiliations:** ^1^School of Tourism and Media, Chongqing Jiaotong University, Chongqing, China; ^2^School of Business Administration, Southwestern University of Finance and Economics, Chengdu, China

**Keywords:** social media, self-presentation, impression management efficacy, scale development, youth group

## Abstract

**Introduction:**

This study seeks to develop and validate a scale for assessing Impression Management Efficacy (IME) in the context of Chinese youth’s self-presentation behaviors on social media. It aims to provide a comprehensive evaluation of their ability and self-efficacy in managing impressions within social media environments.

**Methods:**

For this study, 18 young individuals were selected to participate in in-depth interviews. The specific dimensions of IME were identified through the application of grounded theory categorization. Subsequently, specific measurement questions were formulated by referencing the impression management scale, the social self-efficacy scale, and insights from empirical interviews, leading to the preliminary compilation of the questionnaire. A total of 920 questionnaires were then distributed for a centralized investigation. The collected data underwent repeated testing to refine and finalize the questionnaire.

**Results:**

This study explores the relationship between self-presentation and IME in social media contexts. Through multiple tests and empirical data analysis, IME was delineated into five distinct dimensions: identity management strategy, self-impression management strategy, communication expression efficacy, protection strategy efficacy, and self-presentation efficacy, collectively encompassing 25 measurement items. The study is inherently exploratory in nature.

**Discussion:**

The concept of IME among young people is distinct yet related to traditional notions of impression management and social self-efficacy. While impression management primarily focuses on external behaviors, social self-efficacy assesses one’s perceived social abilities. Both concepts, along with their measurement methods, tend to be relatively singular in focus. IME, however, is closely intertwined with these concepts but possesses a unique conceptual depth and theoretical significance, setting it apart as a multifaceted and nuanced construct.

## Introduction

In the wave of the digital era, social media has emerged as a pivotal platform for daily communication, information acquisition, and self-expression among contemporary youth. This emerging medium not only reshapes people’s communication patterns but also profoundly influences youth’s self-perception, image construction processes, the pursuit of social recognition, and the attainment of social influence (Sundar and Limperos, 2013). As the most active user group on social media, youth engage in various behaviors on these platforms to manage and control their online self-images and expand social relationships. These behaviors encapsulate rich psychological mechanisms and social motivations, warranting in-depth exploration from both practical and theoretical perspectives (Huang and Liu, 2020). At the theoretical level, self-presentation and impression management are core topics in the field of social psychology, with the initial impression management theory focusing on individual behaviors in face-to-face communication contexts. In the context of social media, users participate in social interactions by sharing personal updates or viewpoints through photos, texts, and videos to create favorable impression management (Lu et al., 2024).

Although discussions on user self-presentation and impression management have been progressively deepening in academia, there are still inadequacies in the expansion of related concepts and theoretical exploration. Many studies discuss the influencing factors of self-presentation behaviors or the motivations behind impression management formation (Bareket-Bojmel et al., 2016). Some scholars also argue that there are numerous issues with the measurement of impression management, such as a lack of correspondence with relevant personality factors, absence of manipulation regarding self-impression, discrepancies between self-evaluation and others’ evaluations, unsatisfactory results in cross-cultural validations, and influences from factors like social support (Uziel, 2010). Several scholars have proposed that the refinement of impression management measurements and concepts should not overlook considerations from perspectives such as sense of control and efficacy (Leary and Kowalski, 1990; Uziel, 2010). Therefore, efficacy, as an individual’s subjective evaluation of their own abilities, warrants thorough investigation into how it interacts with youth’s impression management strategies within social media environments. Thus, the Impression Management Efficacy Scale is highly specific, emphasizing individuals’ self-evaluation of their impression management capabilities instead of the behavior itself. This focus aids in comprehending individuals’ confidence in the effectiveness of their own strategies. Furthermore, the perceived efficacy of impression management possesses greater adaptability across diverse cultural contexts and professional settings, enhancing the scale’s universal applicability (Chua and Chang, 2016).

Currently, there is a paucity of research that delves deeply into impression management efficacy, yet this theoretical concept holds significant research value in examining the self-presentation behaviors of Chinese youth on social media. Several questions remain to be explored in depth: What are the psychological motivations behind Chinese youth’s self-presentation on social media? Do their impression management strategies change within social media environments? What are the constituent dimensions of impression management efficacy? Can this scale serve as a self-assessment tool for measuring individuals’ impression management abilities in social media interactions? Based on this, the present study aims to integrate, clarify, and test the concepts related to Chinese youth’s self-presentation behaviors and impression management efficacy on social media, and design a corresponding scale for empirical validation, with the intention of filling this theoretical gap.

## Literature review

### Self-presentation and impression management on social media

Self-presentation, a common behavioral phenomenon in interpersonal communication, refers to the process in which individuals adopt certain strategies for impression management to shape their self-image in daily life (Niu et al., 2015). Self-presentation holds significant importance for individuals in establishing and developing interpersonal relationships. Social media offers diverse self-display functions, allowing users to autonomously select and edit the information they present on these platforms (Wu et al., 2015), enabling strategic or comprehensive self-disclosure (Wright et al., 2017). Relevant research indicates that on social media, everyone tends to showcase the positive and enviable aspects of their lives (Bazarova et al., 2013). To present an appealing picture of their lives, numerous users choose to post content about enjoying delicious meals and traveling activities (Keutler and McHugh, 2022). Simultaneously, to demonstrate a high-quality lifestyle, people also share posts about newly purchased products or luxury items (Lu et al., 2024). Furthermore, to prove that they have good interpersonal relationships, they display scenes of friend gatherings and post received gifts. Driven by the motivation to present a positive self to others, individuals often seek identification from others, particularly paying attention to the comments or the number of likes on their posts after publishing self-presentation-related content (Lee et al., 2014).

With the development of media technology, youth groups engage in self-presentation behaviors on social media to shape impression management (McLean et al., 2015). Self-presentation allows individuals to re-examine themselves, aiming to achieve unity between the subject and the object, serving as a form of self-exploration, display, and presentation. This motivates impression management, where individuals leverage social media and informational content to construct and maintain their personas, thereby garnering likes and comments from others to fulfill the purpose of impression construction (Bij de Vaate et al., 2018). It is evident that self-presentation behavior is closely linked to impression management. So-called impression management refers to the process in which individuals, across different social contexts, control their behaviors and performances to construct or alter the desired impressions held by others and maintain such desired impressions. This process encompasses two stages: impression motivation and impression construction. According to Goffman, individuals’ impression management behaviors typically involve “backstage preparation” and “front stage performance” (Goffman, 1959). His proposed impression management theory employs concepts from dramatic performance theory, aiming to more clearly explain how people control their behaviors and demeanors exhibited in front of others. However, it overlooks the influence of psychological factors in symbolic social interactions and overly emphasizes the role of self-construction in social life, rendering Goffman’s research on impression management inevitably deceptive and defensive in individual self-presentation. As technology evolves, impression management has progressed through periods of face-to-face interaction, online socialization, social media, and interface. Based on Goffman’s impression management theory, people usually desire to control their identity information and image characteristics in social environments (Rodgers, 2015), while self-presentation on social media serves as a crucial means for identity impression management and efficient self-expression (Bayer et al., 2020). When engaging in social interactions on social media, people are highly concerned about the evaluations and opinions of others online, therefore, they make great efforts to construct self-impression management by showcasing a positive self (Jang et al., 2016).

In terms of the relationship between social media self-presentation and impression management efficacy, in general, individuals with higher impression management efficacy may be more confident in using social media for self-presentation. They believe their strategies are effective in influencing others, so they may use social media more frequently and strategically. Individuals may become better at choosing and creating content that resonates, increasing their social status and popularity. More diverse self-presentation strategies are employed, such as creating a positive online image by posting quality content, carefully selected photos, or subtly editing personal information (Bij de Vaate et al., 2018). For example, the interactive style may be more positive and confident, they may respond to comments more frequently, participate in discussions, and be better able to deal with online conflicts, and they are more adaptable to changes in social media platforms and new social norms, thus maintaining the effectiveness of their self-presentation. If individuals lack confidence in their impression management abilities, they may feel social anxiety and tend to reduce self-presentation behaviors on social media to avoid possible negative evaluation (McLean et al., 2015).

### Social self-efficacy in the context of social media

Social self-efficacy, also known as social efficacy, refers to an individual’s self-efficacy in the realm of social interactions. The concept of “self-efficacy” was introduced by American psychologist Albert Bandura, who posited that individuals possess the subjective ability to judge the processes involved in executing relevant behaviors. Subsequently, numerous scholars have argued that self-efficacy represents an individual’s confidence in coping with different environments and challenges posed by new tasks. As this theory has evolved, various types of self-efficacy have emerged, tailored to specific contexts. For instance, scholars have categorized self-efficacy into general self-efficacy, time-related self-efficacy, emotional control self-efficacy, and Internet use self-efficacy, among others (Sherer, 1982 Betz et al., 1996). Initially, Bandura did not present social media self-efficacy as an independent theoretical concept but discussed it as a measurement dimension of self-efficacy. It was not until a study on American college students in 2000 that social self-efficacy emerged as an independent concept for investigation. In this study, social self-efficacy was defined as an individual’s level of confidence in their ability to participate in social tasks, maintain interpersonal relationships, and develop social ties during interactions (Smith and Betz, 2000). Bandura further conceptually defined social self-efficacy in 2001, suggesting that it involves an individual’s belief in their ability to handle matters effectively using their acquired skills or knowledge applicable to social life, or their confidence in receiving positive responses during social interactions. He categorized social self-efficacy into three components: an individual’s confidence in their social skills, their belief in receiving positive feedback in interpersonal interactions, and their application of knowledge about social behavior (Bandura, 2001).

Although social self-efficacy has been addressed within Bandura’s self-efficacy theory, it has not emerged as an independent and deeply researched concept like other types of self-efficacy. Instead, it often exists as a subscale or dimension within a broader self-efficacy or confidence scale. For example, the General Self-Efficacy Scale (GES) includes a social self-efficacy subscale, and the Skill Confidence Scale contains a social confidence subscale. The advent of the Internet era has prompted scholars to focus on types of self-efficacy within online social environments, leading to the introduction of the concept of online social self-efficacy. Aligning with the psychological characteristics of individual development in a networked society, online social self-efficacy has become a new research direction in media psychology. A review of literature on online social self-efficacy reveals that although some scholars have paid attention to it, the relevant literature is not abundant. Several studies have demonstrated that evaluations of social self-efficacy have direct impacts on self-presentation and social psychology. For instance, there is a positive correlation between susceptibility in online communication and social anxiety (Murphy and Tasker, 2011). Social self-efficacy can facilitate self-presentation by alleviating the fear of social evaluation stemming from social anxiety. However, overall, research on the role of online social self-efficacy evaluations in self-presentation is still scarce (Selfhout et al., 2009). Based on the aforementioned discussions, this study defines online social self-efficacy as an individual’s confidence or belief in their ability to successfully initiate, maintain, and develop interpersonal relationships within online social environments.

Through the literature review, this study identifies several deficiencies despite the ongoing research on individual impression management and self-efficacy. Firstly, in today’s landscape of social media development, the conceptual definitions of individual online impression management and self-efficacy are not sufficiently clear. Differences in understanding among scholars regarding their connotations and structures lead to theoretical ambiguities, limiting the depth and systematicness of research. Cross-cultural differences are not clear enough to accurately grasp the impact of impression management efficacy on individual career development and social relations. Secondly, the development of measurement tools is incomplete, lacking unified and effective assessment standards. Additionally, most studies adopt cross-sectional designs with relatively homogeneous methodologies, making it difficult to deeply explore the relationship between individual social media impression management and social self-efficacy. The absence of longitudinal studies also restricts the understanding of their dynamic change processes. On this basis, the relationship between impression management efficacy and other psychological variables (such as self-esteem, social anxiety, leadership, etc.) remains to be explored. Lastly, few studies have developed scales from the perspective of impression management efficacy, nor have they delved into the structure and connotation of impression management efficacy. Therefore, this study develops the Impression Management Efficacy Scale (IMES) from the perspective of impression management efficacy.

### Conceptual dimension construction of “impression management efficacy” based on qualitative analysis

#### Interview design

Due to the scarcity of existing research, identifying the dimensions and underlying factors of impression management efficacy among Chinese youth cannot rely solely on relevant literature studies. Grounded theory, as a qualitative research method, focuses on gaining insights into the essence of phenomena through deep analysis of empirical data and subsequently constructing a systematic theoretical framework based on this. This method encompasses three levels of coding processes: open coding, axial coding, and selective coding. Previous studies have demonstrated that using grounded theory for scale development is an effective approach (Chaudhuri and Holbrook, 2001). In this study, following the grounded procedure proposed by Strauss and Glaser, 1967 dimensions and specific items for measuring impression management efficacy were extracted and determined from the perspectives of impression management and self-efficacy.

This study follows the basic criteria of theoretical sampling to select the research objects, and adopts the method of intensity sampling. In the selection process, focus on those typical cases with high information density and significant degree of variation. The selection of samples is based on the principle of information saturation, that is, whether the samples can contribute new information points to support the construction and further deepening of the theory. The 18 youth interviewees selected for this study include professionals such as teachers, students, doctors, sales personnel, etc. It is helpful to study and understand the commonalities and differences of individuals’ impression management efficacy under different occupational backgrounds. The age range of the interviewees is concentrated between 18 and 35 years, with an average age of 26. They possess good education levels and communication skills, and enjoy documenting their lives through social media, as well as sharing information and interacting with others via social media platforms. The sample covers multiple geographical regions in mainland China, including developed cities and general areas, to reflect individual differences across diverse socio-cultural backgrounds. Additionally, the sample includes participants with varying levels of education and occupational backgrounds, ensuring broad representativeness across key demographic variables. Given that the focus of the study is on self-presentation on social media, particular attention was paid to the participants’ frequency and experience of social media use, ensuring that the sample comprises individuals who are frequent social media users.

According to the “Medium- and Long-Term Youth Development Plan (2016–2025),” the sample composition aligns with the official age range for youth groups (Xinhua News Agency, 2017). There are differences in occupation, education background, and gender among the interviewees, which not only facilitates the discussion of patterns between self-presentation on social media and impression management efficacy but also ensures the diversity and richness of the sample data, providing a solid realistic foundation for this interview. To protect the privacy of the interviewees, their names are replaced with initials, and they are coded as serial number + gender + abbreviated initials of their names. Detailed information of the interviewees is presented in Table 1.

**Table 1:** Basic information table for respondents.

ID	Gender	Age	Occupation	Educational level	Place of residence
1M-SXR	Male	27	Driver	High School	Henan
2F-WJL	Female	20	Student	Bachelor’s Degree	Guangdong
3F-WYW	Female	32	Doctor	Doctorate	Guangdong
4F-LKT	Female	25	Artist	Bachelor’s Degree	Jiangsu
5F-ZJY	Female	31	Doctor	Master’s Degree	Beijing
6M-FJS	Male	28	Journalist	Bachelor’s Degree	Chongqing
7F-SMH	Female	24	Nurse	Bachelor’s Degree	Guizhou
8F-WDN	Female	30	Lawyer	Bachelor’s Degree	Shanghai
9M-FLW	Male	29	Worker	Junior College	Guangdong
10F-LGY	Female	22	Student	Bachelor’s Degree	Gansu
11M-WYH	Male	19	Student	Bachelor’s Degree	Zhejiang
12F-ZYZ	Female	26	Worker	Junior College	Hubei
13F-QWW	Female	22	Student	Bachelor’s Degree	Zhejiang
14F-ZSP	Female	20	Hairdresser	Junior College	Guangdong
15F-NLH	Female	23	Clerk	Bachelor’s Degree	Henan
16F-CFY	Female	30	Engineer	Master’s Degree	Shanghai
17M-ZZH	Male	26	Salesperson	Bachelor’s Degree	Sichuan
18F-LQY	Female	28	Tour Guide	Junior College	Tianjin

This study’s formal interviews spanned 6 months, from January 2023 to July 2023, with each interview lasting for at least half an hour. Given that the interviewees were distributed across the country, the researcher endeavored to contextualize the interviews by asking the participants to refer to their own experiences of self-presentation on social media. The interview outline covered the usage of social media, the behavioral psychology of personal social media self-presentation, individual self-presentation strategies, and motives for impression management. To facilitate data organization, the entire interview process was recorded, and relevant key information was noted. According to the clarity, completeness and coding saturation of the interview data, 18 transcripts of the interview recordings were obtained. In order to avoid such problems as information omission and improper dialect conversion, the researcher and two doctoral students jointly corrected the manuscript after conversion to determine the content of the manuscript. All recorded interviews were converted into formal transcripts within 5 h of the interview, and the transcripts were checked against the recordings. During the research period from May 2023 to August 2023, the research team also regularly observed the social media posts of 12 interviewees to gain insights into their real-life situations. After completing the interviews and observations of the 18 participants, the audio recordings were sorted and summarized in chronological order. During the transcription process, the researcher repeatedly verified the original audio recordings to ensure the accuracy of the primary data. Additionally, research logs were maintained throughout the research period, meticulously documenting the stories of the interviewees.

At the same time, this study uses the built-in qualitative data analysis tools of Nvivo12.0 software, including its coding and classification functions, to systematically and carefully organize, encode and process a large number of collected data, as well as a series of rigorous operation steps such as word frequency statistics. The core purpose of this series of work is to dig deeper and precisely identify the core issues and key points of this research. With the help of Nvivo12.0 software, the process of text encoding becomes more efficient and orderly. The coding rules of this software are used to obtain automatically generated nodes and their sub-nodes, which constitute the basic unit of the initial content analysis text of the interview. Nodes are not only used to distinguish and summarize the data from different sources, but also carry out detailed node division of the text according to the actual situation of the interview content. The setting of sub-nodes further refines these categories, making the analysis level of the text more clear and the structure clearer. There is a distinct hierarchical subordination between the child node and the superior node. This hierarchical structure helps to understand the connotation and extension of the research text more deeply. However, Nvivo12.0 software does not support fully automated analysis of qualitative studies. Its automatic coding function is mainly limited to the recognition of basic elements such as text theme, emotional tendency, name, etc. For deeper text interpretation and analysis, the subjective judgment and intervention of researchers are still needed. During the research process, the research team read literature extensively in related fields to accumulate necessary knowledge reserves. It is also necessary to maintain an objective and neutral attitude in the whole qualitative research process, no matter coding, analysis or classification, to avoid the interference of subjective bias. At the same time, through repeated verification and verification, constantly revise and improve the analysis results, and strive to achieve the objectivity and accuracy of the research results.

#### Coding process

During the open coding process, examine the primary sources meticulously, word for word, dissecting and contrasting the actions, timelines, processes, and various other elements they contain. Subsequently, assign conceptual labels to these elements, converting select pieces of information into nodes, while ensuring that these nodes adhere as closely as possible to the original source data. In the process of research, one ought to maintain a flexible approach, given that the nodes established are merely temporary and open to change, they will undergo further refinement and adjustment as additional data is incorporated. In this particular study, expressions pertaining to self-presentation and impression management were encoded, thereby transforming this information into significant nodes. Furthermore, it is noteworthy that a single paragraph may encompass characteristics of multiple nodes. In this process, 176 pieces of original information were extracted. By carefully and repeatedly comparing these pieces of information, valuable concepts were identified, and the extracted relevant information was conceptually categorized. Ultimately, 18 initial categories were identified and extracted (Table 2).

**Table 2:** Example of interview records and open coding.

ID	Example of interview notes (initial concepts)	Category
1	Caring about others’ opinions mainly stems from wanting to leave a good impression, making them think I’m pretty and positive.	Creating a persona
2	Of course, I want to showcase my strengths and characteristics, but I do not want to overdo the photo editing; I still want to maintain authenticity. Excessive editing can make people dislike you.	Showcasing personal strengths
3	A good selfie can present a favorable self-image, boosting my confidence.	Gaining confidence
4	Everyone loves to see beautiful women; posting pretty pictures lets everyone enjoy them.	Pleasing others
5	When faced with negative comments, I use humor to deflect them; I cannot show negative emotions on my social media.	Using humor to defuse situations
6	The photos I post on social media look better because I’ve edited out any flaws.	Concealing weaknesses
7	I reply to every comment, and I pay special attention to those who usually comment and like my posts. I also comment according to their expectations, both online and in real life.	Analyzing others’ expectations
8	My poses in photos are always proper and dignified, never vulgar.	Acting appropriately
9	Generally, my online language is mild and polite; I do not participate in any online disputes.	Using polite language
10	It’s important to be sincere; you cannot overdo the photo editing, or others will talk badly about you behind your back.	Sense of authenticity
11	I usually choose pleasant words when commenting on others because I hope they’ll do the same for me.	Positive evaluation
12	I always respond to others’ comments promptly; otherwise, it seems impolite, and they might not comment on my posts anymore.	Timely feedback
13	When posting on social media, I usually restrain myself; I cannot just say whatever I want.	Controlling self-presentation
14	Nowadays, I rarely post things like updates on my social media; I do not want others to know about my life.	Minimizing expression
15	I still want my photos to present a positive image, showing everyone a positive and sunny side of me.	Positive image
16	I’m willing to share my status; posting selfies feels like a manifestation of confidence.	Self-confidence
17	When editing photos, it’s important to maintain a realistic appearance; I have a good foundation to work with, and maintaining my true self is crucial.	Maintaining self-identity
18	On social media, it’s important to stay rational and not argue with others.	Rational expression

This study identified initial concepts and abstracted relevant categories from the original interview materials through open coding. During the axial coding process, three experts in the field of media psychology were invited to screen the various categories and measurement items. Subsequently, the research team members conducted a pairwise comparison and comprehensive discussion of the screened results, making slight modifications to the names of the 18 formed categories. Additionally, based on the categorization, some adjustments were made: the categories of “polite language” and “rational expression” were merged into “rational and polite”; since the category of “confidence acquisition” encompassed “self-confidence,” the latter category was removed. Ultimately, 16 categories were retained. These 16 categories were then further classified to extract the main categories and their subcategories. By analyzing the logical relationships among the categories, the 16 categories were ultimately grouped into five main categories (Table 3).

**Table 3:** Main axis coding analysis table.

ID	Main categories	Initial categories	Literature foundation
1	Identity management strategy	Creating a persona	Lyu (2016)
		Showcasing self-advantages	
		Acting appropriately	
2	Self-impression management strategy	Being rational and polite	Leary and Kowalski (1990)
		Controlling self-expression	
3	Sense of efficacy in communication and expression	Pleasing others	Smith and Betz (2000)
		Effective communication	
		Positive evaluation	
		Timely feedback	
4	Sense of efficacy in protective strategies	Using humor to defuse situations	Bolino and Turnley (1999)
		Concealing weaknesses	
		Analyzing others’ expectations	
		Sense of authenticity	
5	Sense of efficacy in self-presentation	Positive image	Sung et al. (2016)
		Gaining confidence	
		Maintaining self	

Selective coding involves integrating categories to refine the core category. This study employed a “causal relationship” coding method to organize and determine the core category, while establishing connections between the core category and other main categories, thereby constructing the dimensions for measuring impression management efficacy. Based on grounded theory analysis, the study can be summarized by the core category of “formation of individual impression management efficacy,” which governs all the data. According to these five dimensions, a scale for measuring impression management efficacy was developed to assess individuals’ self-evaluation of their impression management abilities in social interactions and to explore its constituent elements and scale validation.

#### Theoretical saturation test

A total of 18 interviewees participated in this study, and 18 interview materials were compiled. During the initial data compilation, 15 materials were randomly selected as the original empirical data for the qualitative research process, while the remaining three were reserved for testing theoretical saturation. After conducting coding analysis on the first 15 materials, the remaining three materials were subjected to a new round of coding analysis and category theory construction. Through careful comparison, we found that the resulting category relationships were similar, and no new variables or category relationships emerged. This aligns with the principle of theoretical saturation, making the research conclusions more objective and accurate. Therefore, this study concludes that the indicators of impression management efficacy obtained through grounded theory have reached saturation in both theoretical and empirical materials.

### Conceptualization and scale development of impression management efficacy

#### Conceptualization of impression management efficacy

Based on interview entries and category meanings derived from the grounded process, combined with discussions in relevant literature on impression management and social self-efficacy, this study first confirms the genuine existence of impression management efficacy and distinguishes it clearly from impression management and social self-efficacy. Adopting an emic perspective and focusing on the viewpoint of social media users, this study explores a definition applicable to online interaction contexts, transcending the controversies in social psychology regarding impression management and social self-efficacy. The concept of impression management efficacy is defined as follows: Impression management efficacy arises within specific interaction contexts, reflecting an individual’s ability to effectively employ various impression management strategies in social interactions to shape and maintain a positive image of themselves in others’ minds, along with a positive confidence in their performance in this process. Individual impression management efficacy can influence social performance, predict social anxiety, and facilitate interpersonal relationships.

#### Research tool

The design of the impression management efficacy scale in this paper is carried out with reference to the following research tools. First, in the measurement of impression management, scholars Leary and Kowalski initially divided impression management into impression construction and impression motivation in 1990. It mainly measures “how to change behavior to affect one’s impression in the minds of others” and “the desire of an individual to expect others to form a good impression and perception of oneself,” which is defined by this theoretical framework and concept. Subsequent scholars developed an impression management scale with 9 measurement items based on the measurement methods provided by impression construction and impression motivation (Leary and Kowalski, 1990). Bolino and Turnley (1999) designed the measurement methods of impression management more accurately based on previous relevant studies. The scale divided impression management into acquired impression management and protective impression management (Bolino and Turnley, 1999). Secondly, in the reference of Social Self-efficacy measurement tools, the Scale of Perceived social self-efficacy (PSSE) by scholars Smith and Betz is the most widely used measurement method in the academic world. Designed in 2000, this scale is a single-factor structure applicable to college students and adults. It contains 25 measurement items, involving six dimensions such as social decisiveness, participation in organizing social activities, pursuit of romantic feelings, and making friends with strangers, using 5-level Likert scale. Higher scores indicate greater social self-efficacy (Smith and Betz, 2000).

In the questionnaire design, first of all, the relevant literature and theoretical basis are combed to get the specific concept and connotation of impression management efficacy. Second, building on the conceptualization of impression management efficacy, a crucial task of this paper is to develop its measurement scale. Following a rigorous scale development process, an initial item pool was first constructed. Based on existing measurements of impression management and social self-efficacy in the field of psychology, as well as the qualitative research conducted in this paper, a total of 73 measurement items related to impression management efficacy were obtained. According to the three principles of simplicity, clarity and avoidance of ambiguity, the questions were compiled in the following three forms: (1) the interviewees’ interviews on impression management and social self-efficacy were translated into written language. (2) Adopt or adapt the questions in other impression management and social effectiveness scales that are consistent with the interview content. (3) Compiled according to the dimensions of youth network impression management. According to the above three methods, the initial document items of the “Youth Impression Management Efficacy Scale” were prepared, covering all the secondary codes. Then, the questionnaire was distributed to 15 postgraduate students, and the one-to-one inquiry questionnaire was filled out. In this process, the psychological feelings of the respondents on the measured items during the questionnaire filling can be more accurately grasped. Each item was evaluated, the items with weak differentiation were eliminated, and the title was revised in plain language according to the cultural level of the research object, so as to make it more in line with the reading comprehension ability of Chinese youth. The research team, after three rounds of discussions, revised, deleted, and merged items, resulting in 65 items. Subsequently, four graduate students, as expert social media users, were invited to further streamline the items based on the same principles, leaving 54 items. Finally, we invited three experts to conduct an initial classification and evaluation of the items, including assessing the content validity of the measurement items (such as wording and language), ultimately retaining 49 measurement items.

#### Exploratory factor analysis

Following the scientific steps of scale development, this study initially conducted an exploratory factor analysis on the aforementioned scale containing 49 items to purify the scale and establish the corresponding factor structure. For questionnaire collection, an online questionnaire format was chosen for administration. Considering that convenience sampling facilitates the identification of issues, stimulates new ideas, and forms hypotheses during the research process, and is particularly suitable for the needs of exploratory research, coupled with the fact that this study essentially falls within the scope of exploratory research, the decision was made to adopt a convenience sampling method for sample selection. The scale was designed using a 7-point Likert scale, where 1 represents ‘strongly disagree’ and 7 represents ‘strongly agree’. To minimize potential biases introduced by self-rating, all respondents were thoroughly informed about the purpose of the research (emphasizing anonymous responses and the exclusive use of research results for academic purposes) and were clearly instructed on the matters needing attention when answering, thereby encouraging respondents to complete the questionnaire conscientiously and accurately (Table 4). In the process of exploratory factor analysis, a total of 460 questionnaires were issued to test the index composition of the scale, and the sample population was shown in Table 4.

**Table 4:** Statistics table for basic information of samples in exploratory factor analysis (*n* = 460).

Item	Content	Statistical value	Percentage (%)
Gender	Male	193	41.96%
	Female	267	58.04%
Age	18–23	163	35.43%
	24–29	179	38.91%
	30–35	118	25.66%
Educational background	High school or below	69	15%
	Bachelor’s degree	264	57.39%
	Master’s degree or above	127	27.61%
Place of residence	Urban	347	75.43%
	Rural	113	24.57%

#### Scale purification

This study followed the following methods and steps to purify the initial scale: Firstly, item analysis was conducted to remove items with critical ratios that did not reach significance levels. Secondly, a corrected item-total correlation analysis was performed to eliminate items with low correlation to the total. Subsequently, an analysis of item means and variances was carried out to delete items with extreme values and low variability. Then, exploratory factor analysis was conducted to obtain the specific factor structure. Lastly, a reliability test was administered to assess the measurement’s reliability (Churchill, 1979).

After the aforementioned purification process, a total of 38 items remained in the scale, ensuring that each factor contained 5 or more items. However, considering that some factors still had a relatively large number of items and that some items had loadings below 0.6 on their respective factors, in pursuit of scale simplicity, the study further deleted relevant items within each factor. Following this step, the number of remaining items was reduced to 28. Subsequently, a validity analysis was conducted on the remaining 28 items. This section employed the Kaiser–Meyer–Olkin (KMO) test to examine the scale, with factor analysis only feasible on the basis of good validity. Data analysis revealed a KMO value of 0.89 for the scale, a Bartlett’s Test of Sphericity with *χ*^2^ = 2107.85, df = 241 (*p* < 0.001). A KMO coefficient greater than 0.7 indicates that factor analysis is appropriate, and the approximate chi-square, degrees of freedom, and significance all exhibited good statistical validity, suggesting the presence of common factors among the items and fulfilling the conditions for factor analysis.

The results of the factor analysis indicated that we extracted a total of 5 factors with eigenvalues greater than 1, as detailed in Table 5. These 5 factors collectively explained 77.75% of the variance in the original items, and each factor explained variance largely exceeded 10%. Additionally, the results of the scree plot further supported this conclusion, showing that the scatter points of the first 5 factors were located on a steep slope, while the scatter points of subsequent factors starting from the 6th formed a platform, with their eigenvalues all less than 1. The extraction of these 5 factors demonstrated strong robustness, as the results remained stable and consistent when the data were divided into different subsamples for analysis.

**Table 5:** Table of exploratory factor analysis for impression management efficacy.

Factor	Item	Factor 1	Factor 2	Factor 3	Factor 4	Factor 5	Communality
IMSS	IME5	0.76					0.52
	IME8	0.72					0.46
	IME4	0.62					0.49
	IME6	0.67					0.54
	IME9	0.64					0.47
SIMSS	IME1		0.82				0.61
	IME7		0.76				0.55
	IME2		0.85				0.62
	IME3		0.78				0.53
	IME12		0.73				0.57
CEES	IME15			0.82			0.42
	IME14			0.77			0.40
	IME19			0.74			0.54
	IME16			0.68			0.52
	IME13			0.79			0.49
	IME22			0.72			0.58
PSES	IME26				0.69		0.54
	IME27				0.62		0.46
	IME18				0.71		0.52
	IME20				0.65		0.61
SPES	IME24					0.74	0.53
	IME21					0.80	0.66
	IME17					0.76	0.57
	IME25					0.82	0.64
	IME28					0.70	0.58
	Eigenvalue	16.47	8.34	5.87	5.32	5.15	
	Contribution Rate	21.35	15.76	14.30	13.58	12.92	
	Cumulative Contribution Rate %	20.65	32.82	49.16	63.85	77.75	

For clarity, factors with loadings less than 0.6 are not presented.

Extraction method: principal component analysis.

##### Impression management efficacy overall scale

The Cronbach’s alpha reliability coefficient is 0.90, meeting the “excellent” standard. The results of the sub-dimensions, based on reliability analysis, reveal the following alpha coefficients: 0.88 for identity management strategies, 0.92 for self-impression management strategies, 0.91 for communication expression efficacy, 0.83 for protection strategy efficacy, and 0.86 for self-presentation efficacy. The overall scale’s alpha coefficient is 0.89. Both the overall scale and sub-scales have alpha coefficients above 0.80, indicating good overall reliability of the scale. In summary, the study yielded a 25-item, 5-dimensional Impression Management Efficacy Scale. The identity management strategy, comprising 5 items, primarily describes an individual’s effective management and utilization of their self-identity on social media platforms. The self-impression management strategy, also consisting of 5 items, mainly outlines strategies for actively shaping and maintaining personal image and reputation through content creation and interaction on social media. The communication expression efficacy, composed of 6 items, describes an individual’s confidence and satisfaction felt during communication and expression, reflecting their self-perception of their communication and coping abilities. The protection strategy efficacy, made up of 4 items, focuses on an individual’s evaluation of their ability to protect their online privacy and security. The self-presentation efficacy, with 5 items, primarily describes an individual’s belief in their ability to shape, maintain, and express their personal identity, traits, and values in an online environment.

#### Confirmatory factor analysis

Through the aforementioned exploratory factor analysis, the study derived the formal scale for Impression Management Efficacy. To verify whether the 25 measurement items truly reflect the composition and connotations of the five dimensions, a large-scale questionnaire distribution is required for the adjusted scale. Confirmatory factor analysis will be conducted to further measure the reliability and validity of the questionnaire. Empirical data analysis will be used to test the explanatory power and convergence of each dimension, culminating in the final measurement scale for impression management efficacy.

#### Data collection

In the formal survey, a total of 965 questionnaires were collected. After excluding 45 invalid questionnaires (e.g., haphazard or incomplete answers, short completion times, internal contradictions), 920 valid questionnaires were obtained, yielding a questionnaire qualification rate of 95.3%. The survey was conducted from October 2023 to November 2023. The sample included 347 male respondents, accounting for 37.72%, and 573 female respondents, accounting for 62.28%. The ages of the respondents were concentrated between 24 and 29 years old, with 286 respondents aged 18–23 (31.09%), 419 aged 24–29 (45.54%), and 215 aged 30–35 (23.37%). The demographic variable profile is presented in Table 6.

**Table 6:** Statistical table of basic information for samples in confirmatory factor analysis (*n* = 920).

Item	Content	Statistical value	Percentage (%)
Gender	Male	347	37.72%
	Female	573	62.28%
Age	18–23	286	31.09%
	24–29	419	45.54%
	30–35	215	23.37%
Educational background	High school or below	62	6.74%
	Bachelor’s degree	559	60.76%
	Master’s degree or above	299	32.50%
Place of residence	Urban	618	67.17%
	Rural	302	32.83%

#### Statistical analysis

**Figure 1:**
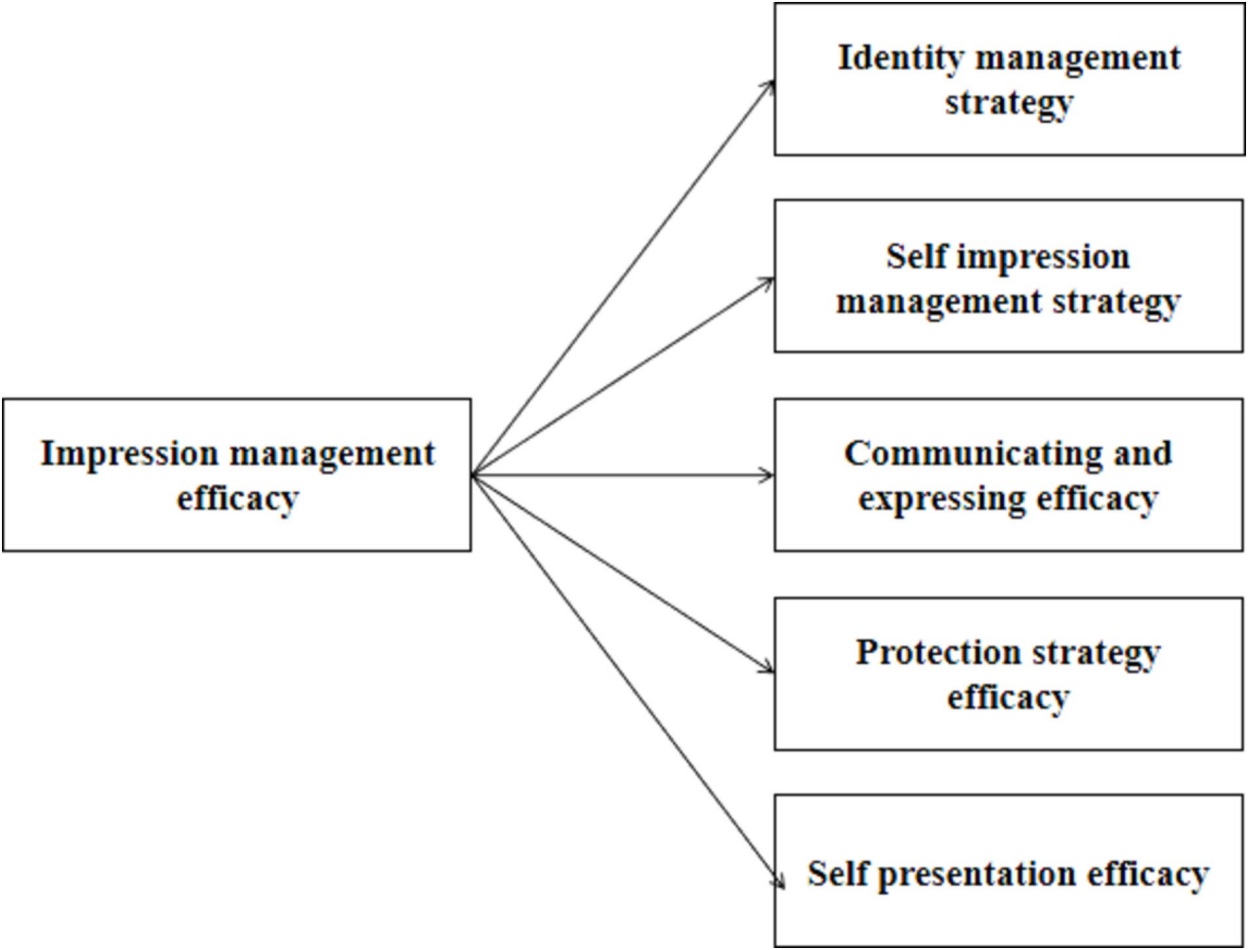
A second-order factor model of impression management efficacy.

Based on the results of theoretical analysis and exploratory factor analysis, this paper proposes that Impression Management Efficacy is a second-order factor structure, wherein the first-order factors consist of five dimensions, including identity management strategies, and the second-order factor is Impression Management Efficacy. The specific model is illustrated in Figure 1.

**Figure 2:**
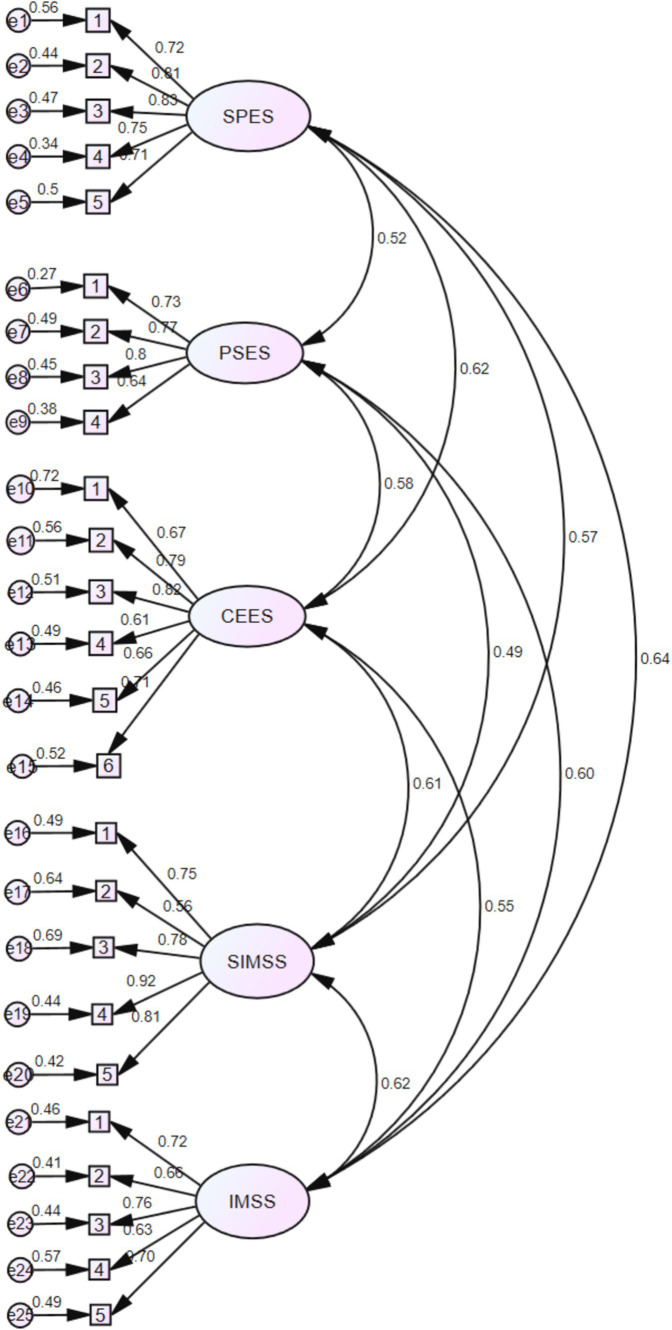
Standardized path diagram of various dimensions of impression management efficacy.

Confirmatory factor analysis tests whether the relationship between a factor and its measurement items aligns with the theoretical relationships designed in the study. Typically, researchers establish a systematic theoretical hypothesis beforehand, proposing that there is a significant correlation between the factor and its measurement items. This verification process often requires validation through structural equation modeling. In this study, there is no formative measurement model due to the large sample size and the lack of complex regulating and mediating variables in the model. Therefore, AMOS 29.0 software was used to conduct confirmatory factor analysis on five dimensions of impression management efficacy. AMOS 29.0 provides a series of model fitting indicators to measure the degree of model fit. These indicators include the Test Comparative Fit Index (CFI), Tuck–Lewis Index (TLI), approximate root-mean-square error (RMSEA), and Chi-square statistics. With a sample size of 920. The model’s confirmatory factor analysis is depicted in Figure 2.

Subsequently, this study will conduct an overall fit test on the dimensions of Impression Management Efficacy using SEM (Structural Equation Modeling) fit indices. Each fit index has corresponding recommended values. For instance, GFI, AGFI, CFI, NFI, TLI, and IFI values greater than 0.90 are indicative of a good fit. The chi-square goodness-of-fit test should yield a *p*-value greater than 0.05, RMSEA should be less than 0.05 (with values less than 0.08 considered acceptable), SRMR should be less than 0.05, and the chi-square to degree of freedom ratio should fall between 1 and 3, all serving as benchmarks for a successful fit. In the SEM model fit indices, the model exhibits a good fit with *X*^2^/df = 1.45 (<3), GFI = 0.90, CFI = 0.93, NFI = 0.94, RMSEA = 0.06, and SRMR = 0.04. The actual values are all within the recommended ranges for a successful fit, indicating a good alignment between the theoretical model and the empirical data. The detailed fit results of the study’s model are presented in Table 7.

**Table 7:** Table of SEM model fit indices.

Indicators	*X* ^2^	*df*	X^2^*/df*	GFI	AGFI	CFI	NFI	TLI	IFI	RMSEA	SRMR
Values	516.20	357	1.45	0.90	0.89	0.93	0.94	0.88	0.87	0.06	0.04

Data source: compiled based on the output results of AMOS equations.

#### Item convergent validity test

Based on non-covariance analysis, it is more scientific to use bootstrapping to explore statistical significance. Bootstrap method was used to calculate the factor load and weight of the measurement model. Bootstrap method is a commonly used test method in SEM model modeling. By sampling the original sample with replacement, it performs the same model estimation for each group of samples re-sampled. Specifically, it refers to uniform sampling with replacement from a given training set. After that, t statistics were calculated with the obtained multi-group parameter estimates to test important parameters in the SEM path model. The aggregate validity of this study was tested through Bootstrap. Convergent validity, also known as convergence validity, typically examines the correlations between items and between factors. Significant correlations indicate good convergent validity of the items. This is often assessed through the Composite Reliability (CR) and the Average Variance Extracted (AVE) in the observed data. A higher CR value (>0.7) indicates greater internal consistency and a higher degree of convergence. The AVE represents the average of the explanatory power of latent variables on observed variables; a higher AVE (>0.5) indicates a higher degree of convergence.

In this study, the sampling times of Bootstrap were set to 5,000 times. As shown in the figure below, the standardized loading coefficients are greater than 0.5, with some reaching 0.7 and 0.8, indicating significant convergent validity for the items related to identity management strategies, self-impression management strategies, efficacy in communication expression, efficacy in protective strategies, and efficacy in self-presentation (*P* < 0.001). The AVE values for each loading path are 0.619, 0.633, 0.559, 0.612, and 0.588, respectively, all greater than 0.5, indicating good convergence of the scale. The combined reliability C.R. values of 0.802, 0.816, 0.864, 0.831 and 0.870 are all greater than 0.7, indicating high internal consistency reliability and good convergence. The specific results are shown in Table 8.

**Table 8:** Validation table of bootstrap aggregation.

Loading path	Unstandardized coefficient	Standardized coefficient	S.E.	C.R. (*t*-value)	*P*	AVE	C.R.
5 ← IMSS	1	0.703				0.619	0.802
4 ← IMSS	1.056	0.634	0.175	7.819	***		
3 ← IMSS	0.837	0.761	0.112	7.243	***		
2 ← IMSS	0.749	0.660	0.096	8.045	**		
1 ← IMSS	0.862	0.724	0.074	7.990	***		
5 ← SIMSS	1	0.809				0.633	0.816
4 ← SIMSS	0.934	0.922	0.079	6.542	***		
3 ← SIMSS	0.815	0.778	0.080	6.153	***		
2 ← SIMSS	0.621	0.561	0.083	7.109	***		
1 ← SIMSS	0.794	0.752	0.074	6.556	***		
6 ← CEES	1	0.712				0.559	0.864
5 ← CEES	1.119	0.660	0.082	5.156	***		
4 ← CEES	0.728	0.609	0.070	6.273	***		
3 ← CEES	0.935	0.822	0.091	6.832	***		
2 ← CEES	0.816	0.791	0.135	6.084	**		
1 ← CEES	0.772	0.673	0.069	7.128	***		
4 ← PSES	1	0.642				0.612	0.831
3 ← PSES	1.215	0.801	0.120	7.758	***		
2 ← PSES	1.014	0.772	0.099	7.480	**		
1 ← PSES	0.949	0.729	0.107	7.754	***		
5 ← SPES	1	0.714				0.588	0.870
4 ← SPES	0.883	0.748	0.136	7.004	***		
3 ← SPES	1.127	0.832	0.103	8.126	***		
2 ← SPES	0.998	0.814	0.097	7.934	**		
1 ← SPES	0.807	0.718	0.124	7.141	***		

**P < <0.01, ***P < 0.001.

#### Criterion-related validity test

Criterion-related validity test, also commonly known as external validation, generally involves first establishing a criterion measure, then determining the criterion measurement, and finally examining the correlation between the data results and the criterion measurement. In this study, the overall Impression Management Efficacy scale was established as the criterion measure, and correlation analyses were conducted with the five dimensions of identity management strategies, self-impression management strategies, efficacy in communication expression, efficacy in protective strategies, and efficacy in self-presentation. The data results revealed significant correlations between the Impression Management Efficacy scale, serving as the criterion measure, and each of the other dimensional measures (*p* < 0.01). The detailed analysis of the criterion-related validity test is presented in Table 9.

**Table 9:** Validation table for criterion-related validity.

	IMSS	SIMSS	CEES	PSES	SPES
IMSS	1				
SIMSS	0.72^**^	1			
CEES	0.66^**^	0.68^**^	1		
PSES	0.61^**^	0.70^**^	0.66^**^	1	
SPES	0.43^**^	0.48^**^	0.41^**^	0.39^**^	1

*P* < 0.01, **P < 0.01.

In summary, the Impression Management Efficacy scale developed in this study has passed the analysis and validation process and is deemed acceptable. This scale consists of 5 dimensions and 25 measurement items, as outlined in Table 10.

**Table 10:** The five dimensions and measurement items of impression management efficacy.

Factor	Measurement item	Content
IMSS	IMSS1	I believe it is very important to manage my online identity effectively.
	IMSS2	I am satisfied with my image on social media.
	IMSS3	I strive to maintain consistency between my social media image and my real-life image.
	IMSS4	I pay attention to and respond to comments and feedback related to my identity.
	IMSS5	I consciously choose which content to share in order to shape my image on social media.
SIMSS	SIMSS1	When presenting myself on social media, it is important to leave a good impression on others.
	SIMSS2	When presenting myself on social media, I hope others perceive me as an outstanding person.
	SIMSS3	When presenting myself on social media, I am able to hide my flaws effectively.
	SIMSS4	When presenting myself on social media, I adjust my presentation based on others’ reactions.
	SIMSS5	When presenting myself on social media, I hope others can see my strengths.
CEES	CEES1	In social media interactions, I can grasp the appropriate time to respond to others.
	CEES2	In social media interactions, I can adopt a suitable tone and attitude.
	CEES3	In social media interactions, I communicate with others in a good attitude.
	CEES4	In social media interactions, I can express myself reasonably.
	CEES5	In social media interactions, I can interact with others in a humorous way.
	CEES6	In social media interactions, I avoid arguments with others.
PSES	PSES1	When evaluating content presented by others, I analyze their expectations.
	PSES2	I can accurately judge which personal information is not suitable for public sharing on social media.
	PSES3	I can remain vigilant when presenting myself, avoiding the disclosure of personal sensitive information.
	PSES4	When faced with negative online evaluations, I can defuse them with a humorous attitude.
SPES	SPES1	In online self-presentation, I have confidence in presenting my true self-image.
	SPES2	In online self-presentation, I have confidence in presenting a positive self-image.
	SPES3	In online self-presentation, maintaining my true self is very important.
	SPES4	In online self-presentation, I usually choose elegant and appropriate clothing.
	SPES5	In online self-presentation, I can maintain confidence and a smiling face.

## Research conclusion and discussion

This study identified the specific dimensions of Impression Management Efficacy through grounded theory categorization and determined the specific concepts, connotations, and operational definitions based on theoretical foundations and interview processes. Subsequently, specific measurement items were established by referencing impression management scales, social self-efficacy scales, and empirical interviews, leading to the initial development of the questionnaire. After multiple rounds of testing and empirical data validation, Impression Management Efficacy was finalized as encompassing five dimensions: identity management strategies, self-impression management strategies, efficacy in communication expression, efficacy in protective strategies, and efficacy in self-presentation, with a total of 25 measurement items. The measurement structure of Impression Management Efficacy was verified through data validation, making this study exploratory.

### Research discussion

#### Identity management strategies of youth groups

The study found that identity management strategies employed by youth groups on social media constitute a series of planned and conscious behaviors and strategies adopted to shape, maintain, and present their self-identity within the social media environment. Interviewees indicated that identity management strategies emphasize how individuals construct and manage their social identity through social media platforms. In reality, the ideal self does not exist in actual life; however, on the virtual stage of social media, individuals can utilize various technical means, identity management strategies, and control mechanisms to present an unrealistic self. When the ideal self-image is uploaded to social media platforms, due to the non-deletable nature of information and the absence or diminution of specific contextual cues during self-presentation, individuals’ self-display on social media gradually exhibits characteristics such as “exhibition” or “display.” This means that once information is posted, it persists and is publicly displayed, detached from its original context, making self-presentation more akin to a continuous, public exhibition. Youth carefully curate and edit the content of their posts, including displays of life photos, personal viewpoints, and emotional states, to actively shape their specific image on social media (Duffy and Chan, 2019). They aim to gain recognition and admiration from peers, communities, and even broader social groups through such self-presentation. In this process, youth flexibly adjust their language and expression based on the audience characteristics and cultural atmosphere of social media platforms to better adapt and integrate into specific social circles, achieving effective identity management. This behavior pattern not only highlights youth groups’ profound cognition and active management strategies of their self-identity but also deeply reflects their psychological needs and social motivations to seek a sense of belonging, identity, and self-worth realization in complex social interactions. Based on this insight, the measurement items regarding identity management strategies in this study, after repeated validation tests, can reflect the identity management strategy capabilities of youth groups to a certain extent.

#### Self-impression management strategies of youth groups

In today’s digital existence, image-based social interaction has gradually integrated into the daily lives of social and cultural practitioners, with the support of media technology empowering youth individuals to exhibit their perfect selves. To continuously maintain a perfect “image” on social media, users must repeatedly consider various aspects such as content, accompanying images, visibility settings, and posting times. Simultaneously, the maintenance process of this “front stage” image is both lengthy and ongoing. Once users successfully construct their ideal image, they often need to meticulously select the content they post to ensure the richness and consistency of their personal image (Marwick and Boyd, 2011). During interviews, respondents repeatedly mentioned their desire to present a self-image that aligns with others’ expectations to ensure that others evaluate them positively and pleasantly. Impression management in self-presentation behavior involves constantly adjusting one’s actions and appearance based on others’ scrutiny and feedback. Therefore, sharing images on social media is crucial for individuals to showcase their strengths and create personal brands. Based on this, the study conducted repeated validation tests on the measurement items of self-impression management, and the research findings also corroborated the dimensional selection of previous measurement methods for impression management (Bolino and Turnley, 1999). In prior research on impression management, individuals’ desire to be seen positively by others or to display their strengths to receive positive evaluations is termed “assertive impression management,” while efforts to weaken self-deficiencies and avoid others seeing a negative self-image are called “protective impression management.” The survey found that “youth container people” living in a fast-paced, high-pressure environment use impression management behaviors and strategies to navigate broader social network relationships. However, it is worth noting that excessive protective impression management fails to create an honest interpersonal atmosphere, and impression management that contradicts good intentions can even worsen social norms.

#### Communication expression efficacy of youth groups

This study found that the concept of communication expression efficacy is closely linked to self-efficacy theory, emphasizing individuals’ beliefs and expectations about their abilities when performing specific tasks. In the specific communication context of social media, youth groups form cognitions about their communication expression abilities through continuous interaction and feedback, which further influence their communication behaviors and strategy choices. This efficacy not only concerns how youth present themselves and establish connections with others on social media but also profoundly affects their influence and voice in online social interactions. Relevant research shows that when individuals improve skills such as language expression, their social efficacy also increases, highlighting the importance of communication expression efficacy in impression management efficacy (Chu et al., 2021). Respondents indicated that good communication and expression skills can enhance self-impression management strategies. In social interaction environments, a good attitude, reasonable expression, and appropriate tone all reflect an individual’s communication expression efficacy. Therefore, measurement indicators for this dimension were designed based on relevant interview data and scale references. In this study, the measurement items for communication expression efficacy included responding to others at appropriate times during social media interactions, using suitable tones and attitudes, expressing oneself reasonably, interacting with others humorously, and avoiding disputes. After multiple exploratory factor analyses, relevant items were deleted and adjusted to obtain the final measurement indicators. The scale exhibits good reliability and structural stability.

#### Protective strategy efficacy of youth groups

On social media, an interactive platform filled with potential risks, youth groups develop cognitions about the effectiveness of protective strategies through practice and learning, which further guides their behavior choices. In previous related research, both impression management strategies and social self-efficacy share the dimension of self-protective strategies. Some scholars have pointed out that triggering self-protective mechanisms in impression management during interpersonal interactions is crucial for maintaining individual participation in social interactions (Tedeschi and Melburg, 1984). According to interview respondents, due to the relatively free environment of social media, it is inevitable to receive negative evaluations when posting photos. Some mentioned using humor to defuse such situations as a way to protect themselves. So-called protective strategy efficacy refers to an individual’s ability to protect their personal safety and information from infringement during social interactions, as well as to resolve any infringements that occur. This is an important dimension of an individual’s impression management efficacy. This indicates that whether an individual has confidence in shaping a good impression in others’ minds also requires maintaining this belief through protective strategies and means (Aizenkot, 2020). The study determined the measurement items for protective strategy efficacy through multiple exploratory factor analyses and reliability and validity tests. The scale also exhibits good reliability and structural stability. The discussion of protective strategy efficacy not only concerns how youth effectively resist the harm of adverse information and protect personal information security on social media but also profoundly influences their coping strategies and psychological resilience when facing risks such as online bullying and privacy leaks.

#### Self-presentation efficacy of youth groups

Self-presentation efficacy refers to the ability to control others’ impressions of oneself by presenting a positive self-image that aligns with personal expectations in social interactions. In the social media environment, users’ self-presentation behaviors mainly encompass the display of personal social information (such as profile pictures, nicknames, etc.) and posting updates. Previous research has shown that self-presentation has two main orientations: one is self-centered, aimed at satisfying self-realization and enhancing self-worth, and the other is other-centered, aimed at satisfying social needs. In the dimension of self-cognition, “self-enhancement” and “self-verification” are considered the two core motivations for people to present themselves online (Bareket-Bojmel et al., 2016). Interview findings revealed that individuals shape impressions in others’ minds through positive self-presentation and build participation and interaction in social networks based on self-presentation. This view supports the strategy of positive and open self-presentation in social networks (Zhao et al., 2020). Through a review of relevant theories and a summary of interview data, five measurement items for self-presentation efficacy were designed, such as confidence in presenting a true self-image, confidence in presenting a positive self-image, maintaining self-identity, choosing tasteful clothing, and maintaining confidence with a smile. After multiple exploratory factor analyses, relevant items were deleted and adjusted to obtain the final measurement indicators. This study’s approach to youth self-presentation efficacy aligns with relevant theoretical logic and measurement indicators, indicating that individual evaluations of self-cognition, appearance, and physical representation are also key factors in impression management efficacy.

#### Theoretical contributions

Firstly, the sense of impression management efficacy among youth groups is a genuinely existent concept, distinct from impression management and social self-efficacy. Focusing on different aspects at the conceptual and hierarchical levels, the sense of impression management efficacy specializes in individuals’ self-perception and confidence during the impression management process, reflecting the psychological motivations behind their impression management endeavors. It encompasses not only impression management strategies but also the performance capabilities and confidence levels exhibited in social situations, as manifested by social self-efficacy (Utz, 2015). Impression management primarily embodies the external expressions of individual self-management behaviors, whereas social self-efficacy evaluates an individual’s social abilities. Therefore, both impression management and social self-efficacy are overly simplistic and one-sided, whether conceptually or methodologically. Consequently, the sense of impression management efficacy is a truly existent concept that is closely related to both impression management and social self-efficacy while possessing its unique conceptual hierarchy and theoretical depth.

Secondly, this study develops and validates a scale to measure the sense of impression management efficacy in Chinese youth’s self-presentation behaviors on social media. By delving into the self-presentation behaviors of Chinese youth, a specific demographic, and the underlying sense of impression management efficacy they exhibit on social media, this research expands the scope of impression management theory and self-efficacy research, making it more aligned with the developmental needs of modern society. It provides a new perspective for studying social media interactions. Current theories and measurements of impression management mostly start from formation motivations, which have numerous issues, such as inconsistencies between self-evaluation and others’ evaluations, and a lack of attention to the context of social media (Uziel, 2010). Especially in the digital society, where individual impression management is closely related to social interactions, there is a need to further refine the measurement of impression management from the perspective of online social self-efficacy. Therefore, this study exploratively designs and validates a scale for the sense of impression management efficacy. Due to the scarcity of research on the sense of impression management efficacy in the current academic field and the absence of accurate research tools for measuring this variable, this study scientifically validates and measures the relevant categories extracted from grounded theory based on related theories and qualitative research. Ultimately, five categories—identity management strategy, self-impression management strategy, communication expression efficacy, protection strategy efficacy, and self-presentation efficacy—are determined as measurement dimensions for the sense of impression management efficacy. The scale development process adheres to specific standards, exhibiting good reliability and validity. It provides a practical and effective tool for measuring youth’s sense of impression management efficacy and lays a foundational tool for subsequent research.

### Research limitations and future directions

Firstly, there are limitations in the sample representativeness and sampling method of this study. Due to constraints in time, personnel, and resources, the study may not adopt a more scientific random probability sampling method but relies on convenience sampling. Although the sample aims to maximize the research scope, it still fails to cover a broader range of Chinese youth groups. Future research can further consider the issue of sample representativeness, adopt stricter sampling methods, and further enhance the external validity of the study, such as through random sampling. Secondly, during the development of the impression management efficacy scale, despite strictly following the procedures and standards of scale design, the selection of scale items, expression methods, and dimension divisions may still be insufficiently objective. Moreover, the self-presentation behaviors of Chinese youth on social media are diverse and complex, with intricate underlying personal psychological motivations. How to comprehensively and accurately capture these behavioral and psychological characteristics poses a significant challenge. Future research should fully consider the presentation of personal information, the choice of language styles, as well as micro-aspects such as individual interaction modes and emotional expressions. Thirdly, the research subjects are Chinese youth groups. In reality, against the backdrop of globalization, youth from different cultural backgrounds may exhibit differences in their self-presentation behaviors on social media. Therefore, future research needs to fully consider the impact of cross-cultural factors on the sense of impression management efficacy and conduct more in-depth cross-cultural comparative studies. Subsequent research can pay closer attention to the influence of the sense of impression management efficacy, as a positive psychological variable, on negative social psychologies such as social anxiety and social burnout. It can also focus on the unique manifestations of the sense of impression management efficacy in social media among elderly groups.

## Data Availability

The raw data supporting the conclusions of this article will be made available by the authors, without undue reservation.
